# Effect of Different Compatibilizers on the Properties of Green Low-Density Polyethylene Composites Reinforced with Bambusa Vulgaris Bamboo Fibers

**DOI:** 10.3390/polym16131760

**Published:** 2024-06-21

**Authors:** Mariane W. Bosenbecker, Eduarda Vieira Silva, Gian Francesco dos Reis Paganotto, Tiago Thomaz Migliati Zanon, Fernanda Langone, Marlon Bender Bueno Rodrigues, Juliano Marini, Jalel Labidi, André Luiz Missio, Amanda Dantas de Oliveira

**Affiliations:** 1 Technological Development Center—CDTec, Postgraduate Program in Materials Science and Engineering—PPGCEM/UFPEL, Federal University of Pelotas—UFPel, Pelotas 96010-610, RS, Brazil; marianebosenbecker@hotmail.com (M.W.B.); ferlangone@gmail.com (F.L.); marlonbueno50@gmail.com (M.B.B.R.); adoliveira@ufpel.edu.br (A.D.d.O.); 2 Department of Materials Engineering—DEMa, Federal University of São Carlos—UFSCar, São Carlos 13565-905, SP, Brazil; tiago.zanon12@gmail.com (T.T.M.Z.); juliano.marini@ufscar.br (J.M.); 3 Chemical and Environmental Engineering Department, University of the Basque Country UPV/EHU, Plaza Europa, 1, 20018 Donostia-San Sebastián, Guipuzcoa, Spain

**Keywords:** bioeconomy, maleic anhydride, tannin, sustainable composites, natural fibers

## Abstract

Low-density green polyethylene (LDGPE) composites reinforced with 5 wt% of bamboo fiber and 3 wt% of a compatibilizing agent (polyethylene grafted with maleic anhydride and tannin) were processed through extrusion and injection molding. Bamboo fiber, Bambusa Vulgaris, was characterized using Fourier-transform infrared spectroscopy (FTIR). The molded specimens were analyzed for their thermal, mechanical, and morphological properties. The estimated concentration was chosen to provide the best mechanical strength to the material studied. FTIR analysis of the fibers revealed the presence of groups characteristic of bamboo fiber and tannin. Differential scanning calorimetry revealed that both compatibilizing agents increased the matrix’s degree of crystallinity. However, scanning electron microscopy (SEM) showed that, despite the presence of compatibilizing agents, there was no significant improvement in adhesion between the bamboo fibers and LDGPE.

## 1. Introduction

The demand for more sustainable alternatives in the manufacture of materials has increased in recent decades, aiming to reduce the negative impacts on the environment and overcome the scarcity of non-renewable resources [1]. Several studies are being carried out with the aim of finding solutions to reduce the use of raw fossil materials and reuse of agroindustry residues, such as the development of biobased polymers and the use of lignocellulosic materials to produce composite materials.

Polymer composites can achieve great properties with low density, replacing several conventional materials. They are formed via a matrix and a dispersed phase, present in smaller quantities to enhance their characteristics [2], and widely used for structural purposes in the automotive, civil construction, bioengineering, and aerospace areas [3]. In this context, green polyethylene, a semi-crystalline polyolefin material developed from ethanol extracted from sugar cane, stands out having the advantage of reducing CO_2_ emissions in the atmosphere since its precursor is a renewable raw material, and it can be recycled at the end of its consumption cycle [4,5]. In addition to the environmental advantages that this new line of polymers offers, it has similar performance in terms of properties, processability, and applications to petroleum-based polyethylene [6,7]. 

Natural fibers have been widely used in composite materials as reinforcement because they have a low cost, are a renewable natural resource, have no toxicity, have good mechanical properties, and are biodegradable and recyclable [8,9]. Fibers can be obtained from jute, sisal, coconut, bamboo, etc. [10], and among them, the use of bamboo from the Bambuseae genus must be highlighted as an alternative in replacing structural materials such as steel and concrete [11], presenting good mechanical properties, lightness, versatility, and a low cost, while being applied in various sustainable composites [12,13].

However, there is a crucial parameter for polymer composites reinforced with natural fibers that must be taken into consideration: interfacial adhesion, which is responsible for the load transfer from the matrix to the fibers [14,15]. Since the polymer matrix generally has hydrophobic characteristics, while the fibers have hydrophilicity, it is necessary to use a coupling agent (compatibilizer) to achieve the chemical mediation between the matrix and the reinforcement improving the adhesion without impairing the mechanical properties of the composite [16,17]. Polymers grafted with maleic anhydride, such as polyethylene (PE-g-MA) and polypropylene (PP-g-MA), are the most widely used compatibilizing agents since these copolymers promote good interface adhesion, resulting in satisfactory properties for the composite when compared to those that are non-compatibilized [18,19]. However, besides being manufactured from petrochemical resources, they have a high energy cost during production and a high price compared to natural raw materials. Therefore, researchers investigate natural coupling agents from lignocellulosic sources for composites, such as lignin and tannin, in order to replace synthetic compatibilizers [20,21,22].

Tannins are water-soluble phenolic compounds found naturally in various parts of plants such as fruits, wood, leaves, bark, and herbs [23]. This compound has antioxidant characteristics and is traditionally used in the leather tanning process and also as flocculants, adhesives, and coatings [24]. Chemically, tannins can be classified as hydrolyzable and condensed, having different applications. Condensed tannins have oligo/polyphenol units containing polar and non-polar parts in their composition, which resembles synthetic compatibilizers, where the apolar aromatic groups have an affinity with the polymer matrix, while the hydroxyl groups interact with the lignocellulosic fibers, anchoring the matrix to the reinforcement agents [25].

In view of the promising properties of tannin for applications as a coupling agent in the production of sustainable composites, this work aims to analyze the compatibilizing effect of polyethylene grafted with maleic anhydride (PE-g-MA) and tannin in bamboo fiber-reinforced, low-density green polyethylene composites.

## 2. Materials and Methods

### 2.1. Materials

The polymer matrix was a low-density green polyethylene (LDGPE), commercially known as SLD4004 resin (I’m green^TM^), supplied by Petroquímica Braskem (Triunfo, Brazil), with a melt flow index of 0.2 g·min^−1^ (190 °C· 2.16 kg^−1^) and a density of 0.923 g·cm^−3^. The bamboo was collected from a bamboo grove in March 2022. It was then cut, dried in an oven at 50 °C, and subsequently ground in a knife mill, and then a 32-mesh sieve was used to control the granulometry of the resulting fibers (BF). Polyethylene-graft-maleic anhydride (PE-g-MA) and tannin (TA) were used as compatibilizing agents. The PE-g-MA was donated by the company Cristal Master (Joinville, Brazil) and has about 1 to 3% of maleic anhydride content, whereas the tannin was supplied by Tanac (Montenegro, Brazil).

### 2.2. Processing of the Composites

#### 2.2.1. Extrusion Compounding

After the raw materials were dried, the composite with 5 wt% of BF (according to the parametrization of other works, the addition of bamboo fiber as a reinforcement of up to 5 wt% improves the mechanical properties by up to 13% [26]) and 3 wt% of compatibilizer (according to the parametrization of other works [27]) were produced using a single-screw extruder (Eco Soluções, Viçosa, Brazil) with L/D of 20. The equipment has four heating/temperature control zones, from the hopper to the die, and the following temperature profile was set: 140 °C (zone 1), 145 °C (zone 2), 150 °C (zone 3), and 190 °C (die). The pure polymer (with no filler) was also processed under the same conditions for comparison purposes. Table 1 shows the mass compositions of the studied composites.

#### 2.2.2. Injection Molding

After the samples’ extrusion compounding, they were granulated and dried. Then, they were submitted to the injection molding process in a mini bench injector, model AXINJET, AX Plásticos Máquinas Técnicas (Diadema, Brazil). The specimens were made to be used in the mechanical tests for tensile strength and Izod impact strength, according to ASTM D-638 [28] and ASTM D-256 [29], respectively.

### 2.3. Characterization of the Composites

#### 2.3.1. Differential Scanning Calorimetry (DSC)

Differential scanning calorimetry (DSC) analyses were performed to investigate the influence of the reinforcing filler and compatibilizers on the thermal properties (melting temperature (T_m_) and crystallization temperature (T_c_)) and degree of crystallinity (X_c_) of the composites. Equipment from TA Instruments (New Castle, DE, USA), model Q-2000, was used, with nitrogen as a carrier gas, at a constant flow of 50 mL·min^−1^. The samples were initially heated from 30 to 200 °C at a heating rate of 10 °C·min^−1^, remaining at this temperature for 3 min to eliminate the thermal history of the samples. After, they were cooled at 10 °C·min^−1^, from 200 to 30 °C for the determination of T_c_ and again heated up to 200 °C at a rate of 10 °C·min^−1^. The degree of crystallinity (X_c_) of the samples was determined using Equation (1) [30].

(1) $$X_{c}=\frac{{\Delta H}_{m}}{{∆H}_{m}^{0}(1-W)}*100\%$$

where X_c_ is the degree of crystallinity (%), ∆H_m_ (J·g^−1^) is the melting enthalpy of the sample, ${∆H}_{m}^{0}$ (J·g^−1^) represents the theoretical melting enthalpy of a 100% crystalline LDGPE, that is, 293 J·g^−1^, and W corresponds to the weight fraction of fiber in the formulation [31].

#### 2.3.2. Uniaxial Tensile Test

Tensile tests were performed according to ASTM D-638 [28] in an Instron Universal Testing System, model 5568, with a load cell of 5 kN and strain rate of 10 mm·min^−1^. At least five specimens were tested per sample.

#### 2.3.3. Izod Impact Strength Test

Izod impact strength tests were performed using Ceast equipment, model RESIL 25 (Instron, São José dos Pinhais, Brazil), according to ASTM D-256. The injection-molded specimens were notched in a Ceast notching machine to a depth of 2.54 ± 0.1 mm. At least eight specimens were tested per sample, at room temperature. In this work, a 5.5 J hammer was used, and none of the specimens suffered total fracture, only partial, which was classified as an NB (non-break).

#### 2.3.4. Scanning Electron Microscopy (SEM)

SEM (Jeol, Tokyo, Japan, model JSM-6610LV) was used to evaluate the matrix-reinforcement adhesion under the use of different coupling agents. Micrographs of the fractured Izod impact strength specimens were obtained. A thin gold layer was deposited onto the material with a metallizer (Bal-Tec, Los Angeles, CA, USA, Mult Coating System MED020).

## 3. Results

### 3.1. Characterization of the Composites

#### 3.1.1. Differential Scanning Calorimetry (DSC)

**Figure 1:**
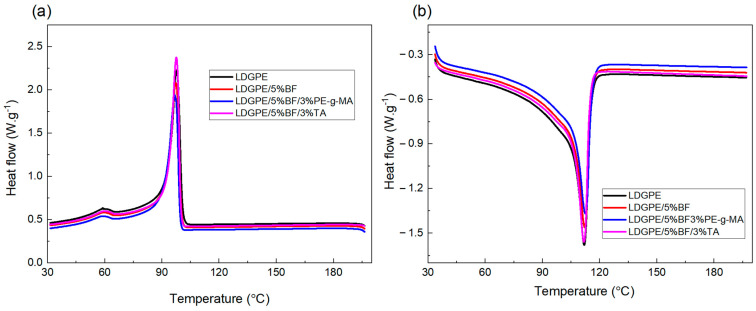
DSC thermograms for the (**a**) cooling and (**b**) second heating of the samples studied.

DSC analysis was used to determine possible crystallinity changes to the matrix after the addition of reinforcement and compatibilizers. DSC thermograms of the cooling and second heating are illustrated in Figure 1, using samples obtained after the extrusion process. The values corresponding to the crystallization temperature (T_c_), crystallization enthalpy (ΔH_c_), melting temperature (T_m_), and melting enthalpy (ΔH_m_) are given in Table 2.

As shown in Table 2, the addition of bamboo fiber and the compatibilizing agents resulted in small changes in T_m_ and T_c_ when compared to LDGPE and a slight increase in T_m_ of the LDGPE/5%BF and LDGPE/5%BF/PE-g-MA composites when compared to the pure polymer. Furthermore, for T_c_, a slight reduction was noted, in which it was considered that the addition of compatibilizers inhibited the growth of LDGPE crystals, which had the effect of forming small and imperfect crystals [21].

It is important to note that the only composite that had a T_m_ lower than that of pure LDGPE was the one with the addition of TA, following trends already portrayed in the literature. In the work developed by Kim et al. [32], the T_m_ and T_c_ of polyvinyl alcohol and hydrothermally treated tannic acid (HTA) composite films decreased as the HTA content increased, related to the increasing disruption in the PVA molecular chains caused by its hydrogen bonding with the HTA. Similar results were found by Liao et al. [33], who observed a decrease in T_m_ in PP and cross–linked tannin (TH) composites before UV accelerated weathering, as the TH content became higher.

When referring to ΔH_m_, a significant decrease in the enthalpy of the composites was observed when compared to the polymeric matrix alone. In general, LDGPE molecular chains can crystallize on their own through an effect known as self-nucleation or via the introduction of a nucleant, an effect known as heterogeneous nucleation [34]. Additionally, the ΔH_c_ also showed a decrease for the LDGPE/5%BF composite compared to the polymeric matrix, and when added, the compatibilizing agents induced different behaviors in enthalpy values in relation to the LDGPE/5%BF composite: while the use of PE-g-MA was responsible for decreasing the enthalpy of crystallization, the TA caused an increase in this value.

In addition, the composites exhibited a progressive decrease in X_c_ when compared with the LDGPE. When comparing the values of crystallinity of the compatibilized and without compatibilizer composites, it can be seen that the incorporation of maleic anhydride and tannin presented decreases in the crystallinity of the materials, attributed to the enhanced adhesion between the LDGPE matrix and the BFs, which disturbs the mobility of polyethylene chains.

#### 3.1.2. Uniaxial Tensile Tests

From the literature, it is known that bamboo fibers present good reinforcement potential for polymeric composites since their unidirectional arrangement provides high stiffness, although their mechanical properties depend on variables such as the type of bamboo and environmental conditions during its growth [35].

**Figure 2:**
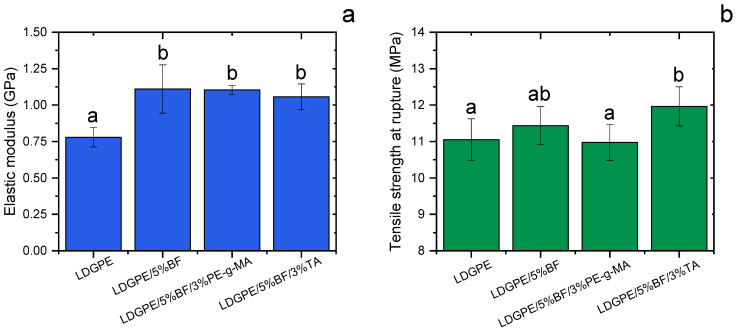
Elastic modulus (**a**) and tensile strength at rupture (**b**) of the LDGPE composites. Values followed by at least one letter in common indicate non-significant distinctions at a 95% confidence (*p* < 0.05) level according to Fisher’s LSD test.

Regarding the elastic modulus (Figure 2a), it can be observed that the composites presented a differentiated behavior when compared to pure LDGPE; it was proven that the bamboo fiber provided a considerable increase in the modulus (~40%). However, similar results were found for the compatibilized composites (LDGPE/5%BF/3%PE-g-MA and LDGPE/5%BF/3%TA) when compared to the uncompatibilized sample (LDGPE/5%BF). This behavior may be an indication that the presence of compatibilizing agents did not improve the interfacial adhesion and/or dispersion state of the bamboo fiber through the LDGPE matrix [36].

For thermoplastic composites, the elastic modulus is generally improved by incorporating a reinforcement because the filler restricts the mobility of the polymer chains and also has a higher stiffness compared to the matrix, contributing to an increase in the stiffness of the composite [22]. It is also believed that the ability of a composite interface to transfer elastic deformation depends on the interfacial stiffness and static adhesion strength [37]. A similar trend was demonstrated in the work of Daramola et al. [38], where an increase in the elastic modulus of high-density polyethylene (HDPE)/bamboo fiber composites was noted when compared to the polymer matrix.

The LDGPE/5%BF and LDGPE/5%BF/3%TA composites showed a slight improvement in tensile strength (Figure 2b). However, for the LDGPE/5%BF/3%PE-g-MA composite, no increase was observed. Meanwhile, as previously discussed, the elastic modulus increased for all composites reinforced with BF, which was related to higher stiffness by other authors [39]. The increase in tensile strength at breaking suggests a small additional degree of interfacial compatibility between the filler and matrix [40], with similar results being found in the work conducted by Salmah et al. [41]. Generally, compatibilized composites with higher crystallinities compared to non-compatibilized composites may result in the improvement of their mechanical properties [42]. In this work, although the crystallinity of the composites was not increased, the tensile strength of the LDGPE/5%BF and LDGPE/5%BF/3%TA composites proved to be the highest found among all the compositions tested (both in the same statistical homogeneous group), elucidating a possible ability of TA to prevent tearing or the breakdown of BF and possibly improving the transfer of mechanical stress between the polymeric matrix and the reinforcing agents.

The addition of tannin could potentially increase the plasticity of the LDGPE/5%FB/3%PE-g-MA composite, suggesting that the tannin could be uniformly dispersed in the polymeric matrix and possibly form stable hydrogen-bonding interactions with the bamboo fiber [43], while its non-polar parts (such as aromatic rings) could interact with the LDGPE [44]. In these scenarios, it is important to highlight that the dispersion of compatibilizers, especially TA, was not investigated in the present research. Furthermore, some studies suggest the ability of compatibilizers to cleave the polymer chain length, causing a decrease in the mechanical performance of the matrix itself [45,46]. Considering this, the low gains obtained in the tensile strength values of compatibilized composites may be due to a weakened matrix.

**Figure 3:**
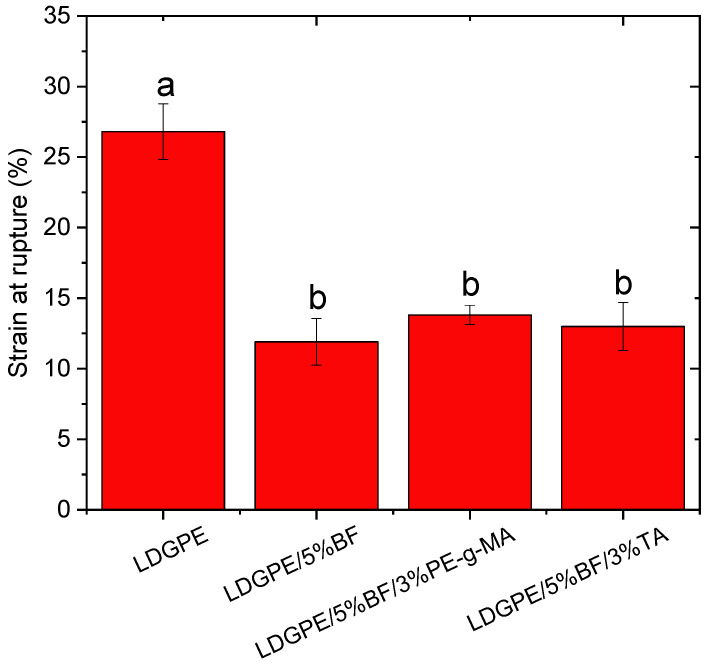
Values of the strain at rupture of tensile strength tests. Values followed by at least one letter in common indicate non-significant distinctions at a 95% confidence level (*p* < 0.05).

Based on the values found in Figure 3, it was observed that the composites exhibited a progressive decrease in the strain at break when compared to the LDGPE, having as a consequence the weak matrix-reinforcement interaction [47], which was unexpected since the strain in the rupture of the reinforced composite and the PE-g-MA- and TA-containing composites was statistically the same. In contrast, the LDGPE/5%BF/3% PE-g-MA and LDBPE/5%BF/3%TA composites showed a slight improvement over LDGPE/5%BF due to the use of compatibilizing agents, which are responsible for providing better interfacial adhesion.

#### 3.1.3. Izod-Type Impact Strength Test

**Figure 4:**
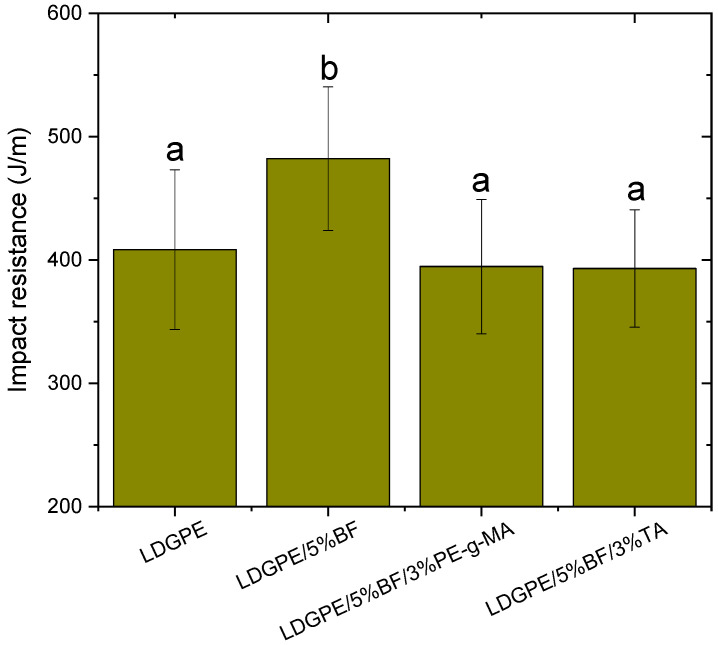
Izod-type impact strength. Values followed by at least one letter in common point to non-significant distinctions at a 95% confidence condition (*p* < 0.05).

The impact strength test is used to measure a material’s toughness, i.e., its ability to absorb energy during impact. The value generated through this analysis is an important measure in the selection of materials for engineering applications. The results of this test can be seen in Figure 4 and Table 3.

The composites showed higher impact energy than the LDGPE. The sample LDGPE/5%BF showed a mean increase of 18% compared to the polymer matrix. The introduction of bamboo fibers promoted an improvement in the impact strength of the composites compared to the matrix; i.e., the energy required to break the samples was higher, proving that the fibers act as reinforcement in the composites [48]. In this work, the composites presented the “fiber bridging” mechanism, in which not all components were fractured during the impact. This happens because part of the load applied to the matrix is transferred to the fibers, which deform, increasing the impact strength of the composite [48]. Similar observations were made by Candido et al. [49], where the authors prepared polyester composites reinforced with sugarcane bagasse.

However, when the compatibilizing agents were added, no significant improvements were observed. This could be explained due to the increased compatibility of the composite after the addition of the PE-g-MA and TA, causing the increased brittleness of the material, which contributes to the composite fracture mode changed from “fiber extraction” to “fiber breakage” [50], in addition to fiber agglomeration [38].

The decrease in impact strength or reduced variation in strength upon the addition of compatibilizer may also be due to the induction of micro-spaces between the fiber and the LDGPE, which generate numerous micro-cracks when impact occurs, enabling crack propagation [13]. It has been noted that PE-g-MA acted as an “anticompatibilizer” in polypropylene composites and that it may have contributed to the pull-out of the reinforcement, as the compatibilizer used was incompatible with the matrix [51]. In the work developed by Yang et al. [21], it was found that, as reinforcement was added, the mechanical properties of the polypropylene/cellulose fiber composites suffered a decrease. This could be explained because the filler used could not be homogeneously dispersed with the tannin. Previous records in the literature report that the molecular weight of the grafted polymer chain, as well as the spacing of the MA units resulting from the grafting, could induce the aforementioned behavior [52,53,54].

#### 3.1.4. Scanning Electron Microscopy (SEM)

**Figure 5:**
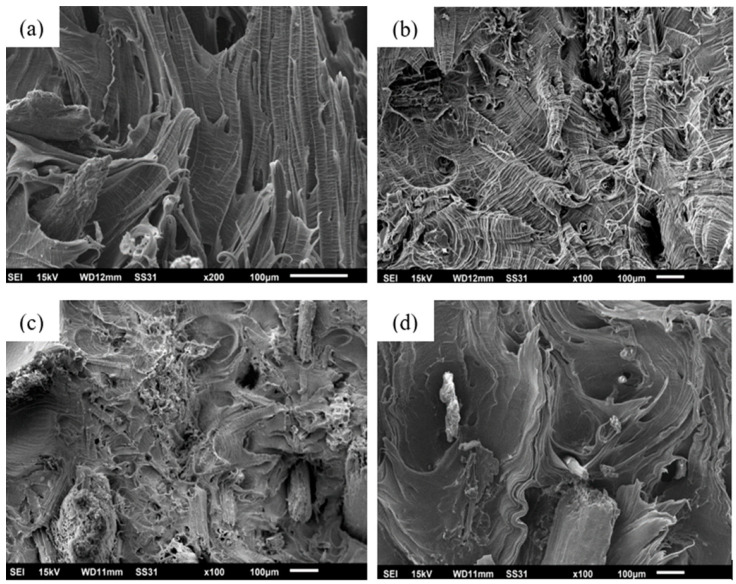
SEM micrographs obtained from the fracture surface of the samples: (**a**) LDGPE; (**b**) LDGPE/5%BF; (**c**) LDGPE/5%BF/3%PE-g-MA; (**d**) LDGPE/5%BF/3%TA.

The morphology of composites is closely related to their properties and particularly to their mechanical strength. As such, changes in the structure of composites can explain the trends that are obtained from their mechanical parameters. Figure 5 shows the SEM micrographs obtained from the fracture surface of the samples after performing the Izod impact strength tests.

As shown in Figure 5a, the fracture surface of LDGPE is comparatively smooth with crack propagation marks [55]. The unstable propagation of cracks is not a consequence of the lack of reinforcement; rather, the lack of reinforcement to control crack growth results in the type of cracks observed. Cracks propagate unstably under external loading, and the main consequence is a lack of reinforcement to control their growth. Although the crack progresses rapidly through the material, there may be a stretching mechanism. This is because this property is directly related to the stress supported and the deformation before rupture [56].

The addition of bamboo fiber in the matrix promotes a significant increase in impact strength, with values of 482.14 J/m. This improvement is fundamentally associated with the ability of the fibers to transfer loads longitudinally, and it can be justified by fracture toughness theory. When the LDGPE/5%BF composite is subjected to impact conditions, several micro-cracks are generated. Consequently, the fibers along these micro-cracks unfold, thus finishing their growth. It was also observed that the presence of short fibers significantly improved the properties related to impact strength [30].

As shown in Figure 5a, the LDGPE has a loose and flaky structure with the presence of microvoids, showing ductile failure at multiple points, which may be related to the fact that its surface is not fully impermeable to moisture and its mechanical integrity is limited in the absence of reinforcement [38].

For the LDGPE/5%BF composite in Figure 5b, several fibers can be described as clustered together, which contributes to a gradual increase in the number of holes and voids, suggesting a ductile fracture. The fibers appear to have separated from the polymer matrix during deformation, which is a clear indication of poor interfacial adhesion [42]. However, the fibers of this material break apart when the composites are broken. This phenomenon occurs due to the good mechanical properties of bamboo fiber, for which more energy is required to break the composites, which results in a higher impact strength [57].

In the work of Daramola et al. [38], it was observed that HDPE composites reinforced with concentrations above 4%-by-weight bamboo fiber exhibited fiber displacement and pullout. The congestion observed for the composites reinforced with 10% by weight means that more load is absorbed via the fibers. The poor adhesion observed for this composite also provides a free path for moisture ingress. The results of Daramola et al. [38] corroborate our findings and reinforce the importance of fiber concentration as a determining factor in failure due to displacement and fiber pullout in HDPE composites. Roumeli et al. [42] found that all HDPE composites reinforced with hemp fibers separated from the polymer matrix during deformation, with a clear indication of lower interfacial adhesion, in addition to voids between the fibers and HDPE.

The failure characteristics shown on the fracture surface of the composites in Figure 5c,d comprise microcracks in the matrix, fiber breakage, and detachment. The micrographs show that the fibers, although pulled out of the matrix (pull-out phenomenon), did not break along with the fracture surface [55]. The pull-out occurrence happened less intensely for the LDGPE/5%BF/3% PE-g-MA composite, in which part of the fibrils of the reinforcement was coated with a polymer. However, for the LDGPE/5%BF/3%TA composite, this phenomenon was more pronounced. This may be closely associated with the structure of this composite consisting of individual microfibrils, which are characterized by forming an irregular fiber surface, and a slight increase in the fiber/matrix interaction area [58]. There was also poor adhesion between fibers and a decrease in impact strength for compatibilized composites and only a slight increase in strength for the LDGPE/5%BF composite [55]. In the study by Anggono et al. [59], it was observed that sugarcane bagasse-reinforced polypropylene composite compatibilized with maleic anhydride showed the displacement and separation of one fiber of the polypropylene, while the fracture of some fibers also occurred.

The LDGPE/5%BF and LDGPE/5%BF/3%TA composites present similar failure characteristics, such as microcracks in the matrix, breakage, and the detachment of fibers (Figure 5c,d). However, in-depth analysis reveals differences in the intensity and distribution of these failure mechanisms between materials. In the LDGPE/5%BF composite, fiber detachment is the dominant mechanism, observed in the separation of fibers from the matrix without their fracture [55]. This characteristic can be attributed to good compatibility between the matrix and the fibers, providing an efficient stress transfer [58].

In contrast, the LDGPE/5%BF/3%TA composite presents a more intense pullout of fibers, evidenced by the partial removal of the fibers from the matrix, accompanying fracture in some cases [55]. This difference is due to the structure of the fibers in this composite, formed via individual microfibrils that generate an irregular surface and increase the area of interaction with the matrix. Furthermore, poor adhesion between the fibers and the matrix, evidenced by a decrease in impact resistance [60], contributes to more pronounced fiber pullout.

Similar studies using polypropylene composites compatibilized with maleic anhydride also demonstrated the displacement and separation of fibers, with the fracture of some of them [59]. Liao et al. [37], in their research, used tannin and lignin as compatibilizers in polypropylene composites and, through results obtained via SEM, observed the poor adhesion of the reinforcement/matrix. In addition, cross-linked tannin particles of irregular shapes were found, confirming the formation of thermosetting tannin particles. Agüero et al. [61], on the other hand, reported that the presence of a significant gap in PLA–flax fiber composites was related to low toughness, and this gap did not allow a good load transfer from the matrix to the fiber. Another factor that may have contributed to the decrease in the related properties is the shear action in single-screw extruders being relatively weaker than in twin-screw extruders, causing less fiber pull-out from the main bundles [62].

## 4. Conclusions

Natural plant-based fibers have a great advantage over traditional synthetic fibers due to their lightweight, non-abrasive, non-toxic, and biodegradable nature. The composites prepared with 3% PE-g-MA or TA did not act as nucleation agents since the crystallinity of the composites did not increase. Mechanical analysis revealed a progressive decrease in the strain at rupture, with the LDGPE/5%BF composite exhibiting the highest impact energy. In tensile strength tests, the LDGPE/5%BF composite showed an improved elastic modulus compared to LDGPE alone, while the LDGPE/5%BF/3%TA composite demonstrated the best tensile strength among all tested compositions. This suggests the potential of TA as a compatibilizing agent in composites. Although the crystallinity of the composites was not enhanced, the LDGPE/5%BF/3%TA composite exhibited the highest tensile strength, indicating TA’s possible role in preventing tearing or the breakdown of BF and enhancing the transfer of mechanical stress between the polymeric matrix and the reinforcing agents. Future work can make valuable contributions to this topic through the development and study of composites blended exclusively with TA, helping uncover the impacts of this natural agent in LDGPE matrices.

## Figures and Tables

**Table 1:** Mass compositions of the studied composites.

Sample	Content (wt%)
LDGPE	100
LDGPE/5%BF	95/5
LDGPE/5%BF/3%PE-g-MA	92/5/3
LDGPE/5%BF/3%TA	92/5/3

**Table 2:** Thermal parameters obtained from the DSC analysis.

Samples	T_c_ (°C)	ΔH_c_ (J·g^−1^)	T_m_ (°C)	ΔH_m_ (J·g^−1^)	X_c_ (%)
LDGPE	97.7	63.4	111.9	69.4	23.7
LDGPE/5%BF	97.3	61.0	112.1	63.6	22.8
LDGPE/5%BF/3%PE-g-MA	96.8	59.3	112.5	63.4	22.7
LDGPE/5%BF/3%TA	97.6	62.9	111.7	62.8	22.5

**Table 3:** Impact strength test values.

Samples	Impact Resistance (J/m)
LDGPE	408.4 ± 64.7
LDGPE/5%BF	482.1 ± 58.2
LDGPE/5%BF/3% PE-g-MA	394.7 ± 54.4
LDGPE/5%BF/3%TA	393.1 ± 47.6

## Data Availability

The study’s data were presented in tables and figures and, if desired, can be obtained by contacting the corresponding author through a reasonable request.
